# On Treatment of Hospital Gangrene, by Circumscribing Wound with Knife

**Published:** 1864-11

**Authors:** T. J. L. De Yampert

**Affiliations:** Surgeon P. A. C. S. Newsom Hospital, Georgia.


ART. V.—*On Treatment of Hospital Gangrene, by Circumscribing Wound with Knife.* By T. J. L. DE YAMPERT, Surgeon P. A. C. S. Newsom Hospital, Georgia.

It is my purpose to offer a few practical remarks upon the treatment of hospital gangrene—the product of thought—confirmed by the test of experience.

Of the pathology of gangrene I might say much that would amount to little, touching the conviction of other minds. I believe "Hospital Gangrene," in its incipiency, to be entirely a local disease; as it advances, however, in different constitutions, at irregular intervals, we mark the traces of constitutional contamination.

This much then said by way of parenthesis, I approach the subject of treatment, the essence of which is expressed in few words.

I advocate the use of fuming nitric acid at irregular intervals, applied locally to gangrenous points; the wound then to be plugged with lint, saturated with ol. terebin; sloughs to be carefully dissected out when sufficiently loosened, and, if indicated, ferruginous or bark tonics, stimulants, etc.

As a medical military man, however, I propose, in addition, a movement in force, so to speak, upon the flank and rear of our enemy—gangrene, and this movement can be accomplished in nearly all cases in their incipiency, without much ultimate loss of structure.

"It is done by circumscribing the wound with a knife."

We have then, at least, a circumference of healthy tissue, an impregnable bulwark of defence.

Now, in the circumference of gangrenous sores, there is a hardened, indurated ring of tissue, which acts as a circular line of defence, behind which much and irreparable damage can be done to structure, while our attention is wholly occupied with the centres of the sore.

I hold that in circumscribing the wound immediately, which has to be repeated afterwards, during progress of cure, seldom more than once, we gain a circular nucleus, as it were, of healthy tissue; which maintains, during the progress of the cure, a disposition to remain healthy; in fact, this now healthy ring of tissue acts as a "whipper in," and confines the disease to the centre.

Phagadena Gangrenosa, in its onward course of destruction, meets with more resistance in muscular and tendinous structure than in does it cellular and adipose tissue, consequently, if we surround and confine the disease, we, in the majority of cases, with facility, will be able to strangle the disease by the gradual encroachment of the healthy ring of tissue radiating towards the centre of the sore.

I have, by this procedure, turned a gangrenous into a healthy sore within twelve hours.

I am confirmed in the belief that this mode of procedure, assisted by the therepeutical agents before mentioned, is the radical and rational treatment for a gangrenous sore. By this plan we will lose less skin, and superficial, as well as deep tissue; the more tissue saved the less liability to contraction, and consequent deformity.

